# Testing Whether Suicide Capability Has a Dynamic Propensity: The Role of Affect and Arousal on Momentary Fluctuations in Suicide Capability

**DOI:** 10.3389/fpsyg.2021.590187

**Published:** 2021-07-27

**Authors:** Keyne C. Law, Michael D. Anestis

**Affiliations:** ^1^Department of Clinical Psychology, School of Psychology, Family, and Community, Seattle Pacific University, Seattle, WA, United States; ^2^New Jersey Gun Violence Research Center, New Brunswick, NJ, United States; ^3^Department of Urban-Global Public Health, School of Public Health, Rutgers University, New Brunswick, NJ, United States

**Keywords:** suicide, acquired capability for suicide, arousal, emotion regulation, affect

## Abstract

To prevent suicidal behaviors, it is crucial to understand the mechanisms and processes that enable an individual to act on suicidal thoughts. Suicide capability, which involves an increased pain tolerance and fearlessness of death, is a critical factor that enables an individual to endure the physical pain necessary to make a lethal suicide attempt. Extant research has largely conceptualized suicide capability as developing linearly in response to painful and provocative experiences, but the emerging literature on the temporal dynamics of suicide has been challenging the notion of linearity in suicide risk. Few studies have directly measured and compared *changes* in suicide capability in response to rumination on different affective states. We sought to experimentally test if rumination in the context of low vs. high arousal emotions will prompt distinct changes in two core components of suicide capability: pain tolerance and fearlessness of death on two undergraduate student samples. In both studies, participants provided measures of subjective emotional state as well as pain threshold, tolerance, and persistence before and after completing experimental manipulations which included both emotion and rumination induction procedures. In the second study, measures of fearlessness about death and physiological arousal (heart rate) were added to the experimental procedures. We found significant decreases in pain threshold, tolerance, and persistence following the experimental manipulations but found no main effects of rumination or suicide risk. These findings suggest that suicide capability can fluctuate but these changes may occur through a different mechanism and/or differ between individuals at varying levels of suicide risk.

## Introduction

Suicide is a worldwide public health issue that claims the lives of ~800,000 individuals annually (World Health Organization, 2014) and demands our attention. Despite the last 50 years of suicide research that has aimed to answer the questions of who, why, and what causes people to die by suicide, our attempts to predict and prevent suicide have not been fruitful (Franklin et al., 2017). Over the past 13 years, suicide rates in the United States (US) have not decreased. On average, the rate of suicide in the United States increased by 1% each year from 1996 to 2006 and grew to 2% per year from 2006 to 2018 resulting in a total increase of 35% altogether (Hedegaard et al., 2020). While there has been a recent shift in researchers' interest in the use of large scale pattern recognition and predictive analytics to predict suicide (Walsh et al., 2017) it is equally important to examine the mechanisms that enable an individual to attempt suicide, allowing us to systematically identify targets for prevention and intervention.

According to two prominent theories of suicide, the Interpersonal Theory of Suicide (ITS; Joiner, 2005) and Three-Step Theory of Suicide (3ST; Klonsky and May, 2015), a critical factor that enables an individual to make a lethal suicide attempt is the capability to endure the physical pain and overcome the fear of death. Indeed, suicide researchers have consistently demonstrated that an elevated risk for suicide is associated with pain tolerance—the maximum level of pain an individual is able to tolerate (Nock et al., 2006; Franklin et al., 2011; Pennings and Anestis, 2013). Specifically, the ability to tolerate more pain has been found to differentiate individuals who have made a suicide attempt from their counterparts who only thought about suicide (Smith et al., 2010). More recent research has also suggested that pain persistence—the difference between the point at which pain is first detected and the point at which an individual can no longer tolerate pain—may also be essential in determining the capability for suicide (Law et al., 2017). In addition to enduring physical pain, attempting suicide requires an individual to overcome their innate fear of death to inflict lethal self-injury. This is supported by existing research on suicide capability, which has found that increased fearlessness of death and dying differentiates individuals who only ideate about suicide from those who have made a suicide attempt (Smith et al., 2010, 2016; Dhingra et al., 2015).

The majority of existing research on suicide capability, however, has conceptualized this variable as relatively stable and increasing in a linear manner in response to painful and provocative experiences (Franklin et al., 2011). Yet, the trajectory of suicide risk seems to be non-linear and fluctuating depending on changes in risk factors. Increasing evidence suggests that suicidal ideation fluctuates from week to week and even from hour to hour and thus appears to be nonlinear (Witte et al., 2006; Bryan and Rudd, 2018; Kleiman et al., 2018). Indeed, the variability of suicidal ideation may be more important than average intensity for predicting future suicide attempts (Bryan et al., 2019). Similarly, suicide capability may also have dynamic propensity such that specific internal and external contexts may momentarily change an individual's ability to make a suicide attempt. The Fluid Vulnerability Theory of suicide (Rudd, 2006) posits that suicide risk fluctuates based on the interaction between predisposing baseline and acute, context dependent risk factors. Suicide capability has been consistently researched as a baseline risk factor for suicide attempts with minimal research examining its potential as an acute risk factor. Existing studies examining pain analgesia during non-suicidal self-injury (NSSI) in individuals diagnosed with borderline personality disorder (BPD) have found that individuals with BPD possess a higher threshold for pain compared to their counterparts without BPD, and this pain threshold is further heightened when they are placed in conditions that elicit high subjective stress (Bohus et al., 2000). Pain threshold has been correlated with aversive arousal in individuals with BPD (Stiglmayr et al., 2001; Ludäscher et al., 2007). Finally, a study using experiential sampling methods found that individuals would report pain analgesia during some NSSI episodes but not others (Selby et al., 2019). Thus, suicide capability may be both a baseline and an acute risk factor for suicide attempts.

In the context of suicide, emotions may be a particularly relevant contributor to momentary fluctuations in the ability to tolerate and persist through pain in order to make a suicide attempt. Notably, a large proportion of psychiatric inpatients who attempt suicide (40.9%) report feeling angry immediately before making a suicide attempt (Chapman and Dixon-Gordon, 2007). Emotions have often been posited to have two qualities: valence and arousal. Valence is defined as the perception of an emotion as being pleasant or unpleasant while arousal is defined as the state of being physiologically activated or deactivated (Barrett, 1998). Past studies have found heightened states of arousal to contribute to the probability of suicide death particularly among individuals with high capability for suicide (Ribeiro et al., 2015). Additionally, physiological differences between negative low arousal affective states (e.g., sadness) and negative high arousal affective states (e.g., anger; Marci et al., 2007) have been found to contribute to differences in pain tolerance (Carter et al., 2002). Specifically, acute experiences of emotions that are of negative valence and high arousal (e.g., anger) have been found to have analgesic effects (Rhudy and Meagher, 2001; Burns et al., 2009). Thus, it is plausible that physiological arousal may moderate the experience of pain and momentarily change suicide capability thereby enabling or disabling an individual's ability to attempt suicide.

All individuals experience a range of emotions, and the experience of negative emotions does not always increase suicide capability. Furthermore, while emotions may have an acute analgesic effect, that effect may not necessarily be sustained long enough for an individual to engage in suicidal behavior. The regulation of negative emotional experiences, related but distinct from the emotional experience itself, may be a crucial factor in understanding suicide risk. Indeed, past studies have found greater emotion dysregulation to increase the desire for suicide and, when paired with in the tendency to engage in painful and/or provocative behaviors (e.g., non-suicidal self-injury), it has also been shown to be associated increases in their suicide capability (Law et al., 2015). Rumination, the repetitive fixation on the experience, causes, and consequences of a negative emotion (Nolen-Hoeksema, 1991), is a maladaptive emotion regulation strategy that has been consistently found to exacerbate and sustain the processing of negative emotion (McLaughlin et al., 2007; Selby and Joiner, 2013). Furthermore, rumination has been associated with increases in both suicidal ideation and suicide attempts (Morrison and O'Connor, 2008; Law and Tucker, 2018). As such, it is plausible that rumination may sustain the analgesic effect of emotion, thereby creating a momentary increase in the ability to tolerate and persist through pain.

Unfortunately, there is a dearth of experimental research testing the stability of suicide capability and examining how different affective states may impact such changes in suicide capability. Existing research on the regulation of emotional states and suicide capability thus far has been limited by the use of descriptive or correlational research designs. As such, we designed these studies to test the differential effects of rumination in the context of a high arousal (anger) vs. low arousal (sadness) emotional state on changes in suicide capability using two undergraduate student samples. We believe that first testing these basic mechanisms in a student sample without substantial suicide history is, practically and ethically, necessary to refine hypotheses and procedures before they are replicated on individuals with significant suicide history who are at greater risk for suicide. We hypothesized that ruminating on experiences of only anger and experiences of anger and sadness together (vs. only sadness) would likely result in a greater increase in suicide capability. Suicide risk, determined by the presence of lifetime suicide ideation, plans and preparatory behaviors, and suicide attempts was also examined as a potential moderator between the aforementioned relationships. Specifically, we hypothesized that individuals who are high on suicide risk will exhibit elevated levels of suicide capability that are comparable across all types of rumination. This would be consistent with existing research that individuals who are high in suicide risk already possess an elevated baseline for suicide capability (Franklin et al., 2011; Ribeiro et al., 2014a).

We also included Heart Rate as a measure of physiological arousal in Study 2. Past studies have supported the theory that a common mechanism exists between pain sensitivity and cardiovascular responses (Vassend and Knardahl, 2004). Particularly, changes in blood pressure and heart rate have been consistently demonstrated to be associated with pain threshold and pain tolerance (Campbell et al., 2006; Duschek et al., 2009). Furthermore, experimental and correlational studies alike have found rumination to be associated with increased blood pressure and heart rate (Ottaviani et al., 2016) and a delayed recovery following cardiovascular reactivity (Glynn et al., 2002). Moreover, the delayed recovery for cardiovascular reactivity can extend past 24 h following the onset of rumination (Ottaviani et al., 2011). Given the association between cardiovascular reactivity, emotion, and decreased pain sensitivity (Appelhans and Luecken, 2008) it is reasonable to anticipate that rumination in the context of different emotional states may impact change suicide capability through arousal as measured by cardiovascular reactivity.

## Methods

### Study 1

#### Participants

Participants who completed the study were 124 undergraduates (M_age_ = 20.86, SD = 8.87; 82.8% female; 65.6% White) enrolled in psychology courses and recruited through the psychology research participation system. Of the 124 participants, 16.9% had thought about suicide in their lifetime, 9.7% have made plans and preparations for suicide, and 4.8% had a history of at least one previous suicide attempt. Detailed demographic information is presented in Table 1.

**Table 1:** Participant demographic information.

	**Control**	**Anger**	**Sadness**	**Combined**	**Full sample**
**Sample 1**					
*N*	32.00	35.00	32.00	23.00	124.00
Age (Mean, SD)	19.97 (2.83)	19.74 (2.83)	22.94 (9.59)	20.91 (5.16)	20.86 (8.87)
% Female	75.00	91.40	75.00	91.30	82.80
**Race**					
% White	62.50	62.90	65.60	73.90	65.60
% African-American	28.10	31.40	28.10	21.70	27.90
% Asian	0.00	0.00	0.00	0.00	0.00
% Hispanic/Latino	3.10	5.70	0.00	4.30	3.30
% Other	6.30	0.00	6.30	0.00	3.30
**Lifetime suicide history**					
% Ideated	9.40	14.30	25.00	21.70	16.90
% Planned	12.50	5.70	12.50	8.70	9.70
% Attempted	3.10	8.60	3.10	4.30	4.80
**Sample 2**					
*N*	25.00	18.00	24.00	17.00	84.00
Age (Mean, SD)	19.84 (2.41)	22.00 (9.99)	20.67 (3.84)	21.47 (4.22)	20.87 (5.51)
% Female	80.00	72.20	91.70	64.70	78.60
**Race**					
% White	56.00	72.20	58.30	70.60	63.10
% African-American	32.00	27.80	29.20	17.60	27.40
% Asian	0.00	0.00	0.00	5.90	1.20
% Hispanic/Latino	4.00	0.00	8.30	0.00	3.60
% Other	8.00	0.00	4.20	5.90	4.80
**Lifetime suicide history**					
% Ideated	20.00	5.60	16.70	35.30	19.00
% Planned	8.00	16.70	4.20	0.00	7.10
% Attempted	0.00	16.70	8.30	5.90	7.10

#### Procedures

Upon registration for the study, a secure link was sent to the participants directing them to the online phase of the study. After reviewing the informed consent form and consenting to participate in the study, participants were asked to complete a battery of online questionnaires focused on demographic variables and trait measurements of psychiatric variables such as their history of suicidal ideation and suicide attempts. They were then randomly assigned to receive instructions to a control condition where they described the room they were in or an experimental condition where they provided a narrative describing an event that made them feel (a) Anger Only, (b) Sadness Only, or (c) Anger and Sadness Combined using the Pitman Protocol (Pitman et al., 1987). Participants who did not provide appropriate narratives that contained sufficient detail (>250 words) for the emotion induction procedure were excluded from participation in the laboratory phase of the study. Between their participation in the online phase and the laboratory phase of the study, the narrative provided by each participant was written into a script and recorded into an audio file to increase immersion into the personalized imagery task used for the emotion induction procedure.

In the laboratory session, participants completed an interview assessing suicide risk, a self-report measure of baseline subjective emotional state, and a cold pressor task (CPT) to measure baseline levels of pain threshold, tolerance, and persistence. In order to minimize potential of third variable effects on pain tolerance variables participants were asked to refrain from taking analgesics (e.g., aspirin, acetaminophen) and other pain suppressants for at least 8 h (Bender et al., 2012), and ingesting sugared foods and alcoholic beverages for at least 1 h prior to their scheduled appointment (Mercer and Holder, 1997).

Participants were then guided through a personalized idiographic emotion induction using the audio file recorded from the narrative they provided in the first stage of the study and subjective emotional state following the emotion induction procedure was measured. Subsequently, participants were visually guided through the rumination induction procedure (Nolen-Hoeksema and Morrow, 1993), which was followed by another measure of subjective emotional state. They completed the CPT a second time to test for changes in state pain threshold, tolerance, and persistence. Finally, subjective emotional state was measured again at the end of the study. Suicide risk was assessed at the end of the study as a means to ensure the participants' safety after leaving the laboratory. Participants were also debriefed and provided with coping skills and local/national counseling services. All self-report questionnaires in the laboratory session were completed on laboratory computers. Suicide risk assessments and CPTs were administered by trained research assistants. The current study protocol was approved by authors' Institutional Review Board.

#### Measures

##### Self-Injurious Thoughts and Behaviors

Suicide risk was determined at baseline by the presence of lifetime suicidal ideation, plans and preparations, and attempts assessed using the Self-Injurious Thoughts and Behaviors Interview (SITBI; Nock et al., 2007). The SITBI is a structured interview which assesses the presence, age of onset, frequency, and severity of suicide related thoughts and behaviors, such as suicide attempts, gestures, plans, ideation, and NSSI. For both studies, individuals who reported no history of suicidal ideation, plans, and attempts were coded with a suicide risk rating of 0; those who have engaged in suicidal ideation only were coded with a suicide risk rating of 1; those who have engaged in plans and preparatory behaviors were coded with a suicide risk rating of 2; and finally, those who had previously made a suicide attempt was coded with a suicide risk rating of 3. In past studies, the SITBI has demonstrated strong inter-rater reliability and test-retest reliability, as well as strong concurrent and convergent validity (Nock et al., 2007).

##### Subjective Emotional State

The Positive and Negative Affect Schedule (PANAS; Watson et al., 1988) was used to evaluate the subjective emotional state of participants at baseline, after the emotion induction procedure, and after the rumination induction procedure. Participants provided ratings on 10 positive emotion items and 10 negative emotion items, which represented how they were feeling “right now, at the present moment” using a 5-point scale where 1 (not at all or very slightly) and 5 (very much). The PANAS has shown good test-retest reliability in past studies using a sample of students (Watson et al., 1988) as well as good convergent validity (Mackinnon et al., 1999). In the Study 1 sample, both the positive (α = 0.72–0.79) and the negative (α = 0.68–0.82) affect scales of the PANAS demonstrated fair internal consistency. In the Study 2 sample, both the positive (α = 0.90–0.94) and the negative (α = 0.84–0.89) affect scales of the PANAS demonstrated good to excellent internal consistency.

##### Baseline and State Pain Experiences

The cold pressor test (CPT) was used to examine participants' pain threshold and ability to tolerate and persist through pain past the pain threshold. The CPT is a frequently used pain induction procedure in studies examining NSSI (Bohus et al., 2000; Gratz et al., 2011). Participants were asked to submerge their hand, up to their wrist, in a cooler containing a mixture of water and crushed ice maintained at 2°C with a water circulator that prevents the water surrounding the participant's hand from warming. These procedures are consistent with previous studies that have used the CPT to measure pain tolerance in the context of suicide and self-injury (Franklin et al., 2011; Ammerman et al., 2017).

Participants were asked to alternate hands (dominant/non-dominant) between the first trial and the second trial; furthermore, hand order was counterbalanced across trials. Time elapsed was measured and recorded using two timers which both began when the participant's hand was submerged and stopped at pain threshold and pain tolerance, respectively. The time at which participants first indicated pain was recorded as their Pain Threshold. Pain Tolerance was operationalized as the time elapsed until the participants pulled their hand out of the water and indicated that they could no longer tolerate the pain. Finally, Pain Persistence was recorded as the time elapsed between the participant's Pain Threshold and Pain Tolerance. A 2-min time limit was used for the task to reduce outliers as past studies have found that participants seldom continue past 2 min and those that do often continue due to a numbed sensation in their hand (Franklin et al., 2012).

#### Experimental Manipulations

##### Emotion Induction

An adapted version of the Pitman Protocol (Pitman et al., 1987) was used to induce the emotional contexts in which participants were told to ruminate. In the online phase of the study, participants were instructed write about a situation in which they felt sad or angry and to include specific details about the sequence of events, people involved, context, descriptions of thoughts, feelings, and physical reactions that were experienced. They were then asked to select the bodily sensations and emotions they experienced during the event from two separate lists. Finally, they listed the thoughts that they were experiencing during the situation they described. The information acquired from the participant were combined and written into scripts between 350 and 550 words in length and subsequently recorded into 2-min audio files using simple, direct language in the active voice and in the second person. The audio file was presented to the participant in the experimental session. Participants who did not provide enough detail (e.g., <250 words) in their narratives to effectively elicit emotion as part of the emotion induction procedures were not invited to participate in the laboratory phase of the study.

##### Rumination Induction

To induce rumination, the rumination induction protocol developed by Nolen-Hoeksema and Morrow (1993) was adapted, by changing verb tenses, to guide participants to think about their emotional state, within the context of the event they heard during the emotion induction. Participants were delivered 45 items (e.g., “think about why people treated you the way they did,” “think about why you reacted the way you did.”) through a series of slides over the course of 8 min.

### Study 2

#### Participants

Participants for this study were 82 participants (M_age_ = 20.87, SD = 5.51; 78.6% female; 63.1% White) enrolled in psychology courses and recruited through the psychology research participation system. Of the 82 participants, 19.0% had thought about suicide in their lifetime, 7.1% had made plans and preparations for suicide, and 7.1% had a history of at least one previous suicide attempt. Past literature examining the role of emotion and rumination on cardiovascular activity had yielded effect sizes in the large range (Vassend and Knardahl, 2004; Ottaviani et al., 2011, 2016). Detailed demographic information is presented in Table 1.

#### Procedures

Study 2 directly replicated and extended upon Study 1 with the inclusion of measures of fearlessness of death and cardiovascular reactivity. Specifically, participants were connected to the BN-RSPEC wireless transmitters and receivers and the Biopac MP150 Data Acquisition System. Three pre-jelled electrodes were allowed to warm on the participants' skin as the initial suicide risk assessment was administered to improve the integrity of the acquired physiological data. After the initial visual inspection of the participants' physiological data and necessary adjustments were made, baseline measurements of the participants' emotional state and resting heart rate were taken. A measure of subjective fearlessness about death and the CPT was administered to measure baseline levels of suicide capability. Following the first CPT, Participants received an idiographic emotion induction, based on the narrative they provided in the online stage of the study using the Pitman Protocol (Pitman et al., 1987) in the form of an audio recording. They were then asked to rate their subjective emotional state following the emotion induction procedure. Subsequently, participants were visually and audibly guided through the rumination induction procedure (Nolen-Hoeksema and Morrow, 1993) followed, again, by a measure of subjective emotional. Subsequently, participants provided another measure of their fearlessness about death and completed the CPT again to test for changes suicide capability following the experimental manipulations. Heart rate was measured during both cold pressor tasks as well as the emotion and rumination induction tasks. Finally, after a recovery period of ~20 min, another measurement of the participants' heart rate and subjective emotional state were taken. A final risk assessment was administered and participants were debriefed before their participation in the study was complete. All self-report questionnaires and experimental manipulations in the laboratory session were delivered using laboratory computers. Behavioral (CPT) and physiological (HR) measurements were recorded by trained research assistants.

#### Measures

##### Self-Injurious Thoughts and Behaviors

Suicide risk was determined at baseline by the presence of lifetime suicidal ideation, plans and preparations, and attempts assessed using the Self-Injurious Thoughts and Behaviors Interview (SITBI; Nock et al., 2007).

##### Subjective Emotional State

The Positive and Negative Affect Schedule (PANAS; Watson et al., 1988) was used to evaluate the subjective emotional state of participants at baseline, after the emotion induction procedure, and after the rumination induction procedure. In the Study 2 sample, both the positive (α = 0.90–0.94) and the negative (α = 0.84–0.89) affect scales of the PANAS demonstrated good to excellent internal consistency.

##### Baseline and State Pain Experiences

The cold pressor test (CPT) was used to examine participants' pain threshold and ability to tolerate and persist through pain past the pain threshold. Procedures for the CPT were directly replicated from Study 1. In study 2, however, the cooler and water circulator that was used in Study 1 was replaced by an Anova A-40 Refrigerated Circulator System.

##### Cardiovascular Reactivity

A measure of cardiovascular reactivity was added to the laboratory phase of study 2. Specifically, we assessed for changes in Heart Rate (HR) derived from electrocardiogram (ECG) acquired using the Biopac MP150 Data Acquisition System and the BN-RSPEC wireless transmitters and receivers. Data were recorded through Acqknowledge 4.4.2 using a sampling rate of 1,000 samples per second. Pre-jelled electrodes were placed below the participants' right and left clavicles and on the left iliac fossa. Measurements were taken at 10 time points including baseline, during both sets of experimental manipulations and both cold pressor tasks, and after a 20 min follow-up recovery period. Physiological measurements that were not task-related (e.g., baseline, post-recovery) were measured using 300 s periods. In preparation for data analysis, all ECG waveforms were visually inspected for noise and heartbeats were identified using QRS peak detection.

##### Fearlessness of Death

In study 2, the 7-item Acquired Capability of Suicide Scale—Fearlessness About Death (ACSS-FAD; Ribeiro et al., 2014b) was included to measure fearlessness of death before and after the experimental manipulations. Participants responded to items using a 5-point Likert Scale where 0 (not at all like me) and 4 (very much like me). Scores on this scale range from 0 to 28 with higher scores indicating greater levels of fearlessness about death. In past studies, the ACSS-FAD has demonstrated adequate internal consistency as well as convergent validity with self-report measures assessing fear of suicide and the courage to attempt suicide (Ribeiro et al., 2014b). In the Study 2 sample, the ACSS-FAD demonstrated poor internal consistency (α = 0.45–0.49) and results examining changes ACSS-FAD are uninterpretable and excluded from this report.

#### Experimental Manipulations

The experimental manipulation procedures used in Study 2 were directly replicated from Study 1 with the addition of an audio recording where each item of the rumination induction protocol were read aloud with their with corresponding text in visual slides.

### Data Analytic Procedures

#### Subjective Emotional State and Manipulation Check

To determine if the emotion and rumination inductions produced the intended effect on the participants, a 4 (Time: Baseline vs. Post-Emotion vs. Post-Rumination vs. Recovery) X 4 (Neutral vs. Anger Only vs. Sadness Only vs. Anger and Sadness) repeated measure ANOVA (RM-ANOVA) and subsequent Bonferroni-corrected pairwise comparisons were used to test for main and interaction effects of Time and Condition on subjective emotional state (positive affect subscale, negative affect subscale, sad item, anger item). Based on previous studies using similar forms of experimental manipulations (Rusting and Nolen-Hoeksema, 1998; Ciesla and Roberts, 2007; Wisco and Nolen-Hoeksema, 2009), a significant increase in negative affect and items relevant to the assigned Condition (anger and sadness) between baseline and post-emotion induction was expected. It was also predicted that a significant increase between post-emotion induction and post-rumination induction would be observed. Finally, negative affect and items relevant to the Conditions were expected to decrease and return to baseline between post-rumination induction and at the end of the laboratory session. The opposite effects were anticipated for positive affect.

#### Primary Analyses

To test the aforementioned hypotheses, we had planned on using a series of hierarchical regression analyses but upon further consideration decided to use a Linear Mixed Model (LMM) using SPSS instead given that LMM will allow us to specify random effects and explicitly partition the variance associated with these differences instead of incorporating them into the general error term. For both Study 1 and Study 2, three separate models were used to test whether or not anger rumination would lead to greater increases of state Pain Threshold, Tolerance, and Persistence compared to sadness rumination. Study 1 Descriptive statistics and correlations can be found in Table 2. Study 2 descriptive statistics and correlations can be found in Table 3. Condition, Suicide Risk, change in pain responses between Baseline and Post-Manipulation (Time), as well as their interactions were entered into the model as fixed effects. The Repeated measure of Time on each individual participant was also entered into the model as a random effect.

**Table 2:** Study 1 correlations and descriptive statistics.

	**1**	**2**	**3**	**4**	**5**	**6**	**7**
1. Suicide risk	1						
2. T1 threshold	−0.13	1					
3. T2 threshold	−0.18^*^	0.79^**^	1				
4. T1 tolerance	−0.15	0.58^**^	0.56^**^	1			
5. T2 tolerance	−0.18^*^	0.54^**^	0.64^**^	0.76^**^	1		
6. T1 persistence	−0.10	0.05	0.15	0.84^**^	0.57^**^	1	
7. T2 persistence	−0.15	0.17	0.24^**^	0.70^**^	0.82^**^	0.75^**^	1
Mean	0.51	16.99	13.19	41.49	32.42	24.52	20.62
SD	0.86	17.09	14.20	31.13	28.56	25.32	22.68
Min	0.00	0.00	1.00	4.00	1.00	0.00	0.00
Max	3.00	119.00	119.00	124.00	121.00	109.00	107.00

*T1, Baseline; T2, Following Experimental Manipulations*.

* *Correlation is significant at the 0.05 level*,

** *Correlation is significant at the 0.01 level*.

**Table 3:** Study 2 correlations and descriptive statistics.

	**1**	**2**	**3**	**4**	**5**	**6**	**7**	**8**	**9**	**10**	**11**	**12**
1. Suicide risk	1											
2. T1 threshold	−0.01	1										
3. T2 threshold	−0.05	0.65^**^	1									
4. T1 tolerance	0.19	0.66^**^	0.60^**^	1								
5. T2 tolerance	0.19	0.59^**^	0.76^**^	0.80^**^	1							
6. T1 persistence	0.24	0.38^**^	0.46^**^	0.94^**^	0.73^**^	1						
7. T2 persistence	0.32^*^	0.44^**^	0.48^**^	0.75^**^	0.92^**^	0.74^**^	1					
8. T1 fearlessness	0.02	−0.04	−0.03	0.03	0.09	0.07	0.14	1				
9. T2 fearlessness	−0.02	0.04	0.11	0.03	0.11	0.02	0.05	0.84^**^	1			
10. Resting HR	0.03	−0.08	−0.11	−0.14	−0.02	−0.15	0.02	−0.12	−0.07	1		
11. CPT1 HR	0.05	−0.05	−0.19	−0.09	−0.09	−0.09	−0.01	−0.17	−0.10	0.58^**^	1	
12. CPT2 HR	0.12	−0.08	−0.15	−0.15	−0.07	−0.14	−0.02	−0.13	−0.04	0.55^**^	0.74^**^	1
Mean	0.55	11.45	9.74	33.02	26.14	21.83	16.77	12.79	12.66	80.83	93.15	89.26
SD	0.91	7.94	7.47	22.93	18.80	18.64	14.29	4.81	4.93	10.27	10.65	10.48
Min	0.00	0.00	0.00	0.00	0.00	0.00	0.00	0.00	0.00	57.57	68.08	61.21
Max	3.00	44.00	36.00	115.00	93.00	104.00	77.00	23.00	24.00	103.97	127.18	117.43

*T1, Baseline; T2, Following Experimental Manipulations*.

* *Correlation is significant at the 0.05 level*,

** *Correlation is significant at the 0.01 level*.

#### Secondary Analyses

We used Model 4 in PROCESS (Hayes, 2013) with 10,000 boot strapped samples to test if the Multi-categorical Condition would have an indirect effect on suicide capability through changes in heart rate. Given that we are most interested in the effect of arousal on changes in pain responses from the first cold pressor task to the second cold pressor task, we used the difference between heart rate during the first cold pressor task and heart rate during the second cold pressor task as the mediating variable. Simple Indicator coding was used to compare each experimental condition with the Control Condition.

## Results

### Covariate Selection

To determine an appropriate list of covariates, we used a series of analyses of variance (ANOVAs) to determine the influence of Race and Sex on changes in pain threshold, tolerance, and persistence. We then examined zero-order correlations to determine if Age and Trait Rumination, as measured by the Ruminative Response Scale (RRS; Treynor et al., 2003), were associated with changes in pain threshold, tolerance, and persistence. In Study 1, Sex was associated with changes in pain tolerance [*F*_(1, 121)_ = 5.76, *p* = 0.02] but not pain threshold or persistence (all *p*s > 0.10). There were no significant effects of Race on changes in pain responses (all *p*s > 0.16). Neither Age (all *p*s > 0.58) or Trait Rumination (all *p*s > 0.16) were correlated with changes in pain responses. As such, Sex was included as a covariate in the primary analyses examining pain tolerance.

In Study 2, there was a significant effect of Sex on changes in pain tolerance [*F*_(1, 61)_ = 7.23, *p* = 0.009] and pain persistence [*F*_(1, 61)_ = 16.04 *p* < 0.001] but not pain threshold (*p* = 0.31). There was also a significant effect of Race on changes in pain persistence [*F*_(2, 60)_ = 5.17, *p* = 0.008] but not pain threshold or tolerance (all *p*s = 0.45). Neither Age (all *p*s > 0.44) or Trait Rumination (all *p*s > 0.20) were correlated with changes in pain responses or arousal. As such, Sex was included as a covariate in the primary analyses for pain tolerance and persistence. Similarly, Race was included as a covariate in the primary analyses examining pain persistence.

### Subjective Emotional State and Manipulation Check

In the Study 1 sample, we found significant main effects of Time [*F*_(2.05, 208.18)_ = 3.547, *p* = 0.030] on changes in Positive Affect such that there was a significant decrease in positive affect from rumination (M = 1.55, SD = 0.49) to recovery (M = 1.44, SD = 0.47; *p* = 0.009). There were no other significant main or interaction effects on positive affect (all *p*s = 0.10 = 0.74). In terms of Negative Affect, we found a significant main effect of Time [*F*_(2.66, 266.27)_ = 7.89, *p* < 0.001] and a significant 2-way interaction between Time and Suicide Risk [*F*_(7.99, 266.27)_ = 2.997, *p* = 0.03]. Specifically, individuals with no history of suicidal ideation, plans and preparations, and attempts exhibited a significant decrease in negative affect between the emotion induction (M = 1.98, SD = 0.63) and rumination induction task (M = 1.79, SD = 0.58, *p* < 0.001) as well as a significant decrease in negative affect following the rumination induction task at recovery (M = 1.65, SD = 0.53, *p* = 0.002). Participants who have previously made plans and preparations for suicide also exhibited a significant decrease in negative affect between the rumination induction task (M = 1.89, SD = 0.40) and recovery (M = 1.40, SD = 0.26, *p* < 0.001).

In the Study 2 Sample, we found significant main effects of Time [*F*_(2.27, 154.59)_ = 7.747, *p* < 0.001) on Positive Affect such that, compared to baseline (M = 2.62, SD = 0.99), there was a significant decrease in positive affect after the emotion induction (M = 2.10, SD = 0.90, *p* = 0.001) and this decrease was maintained following the rumination induction (M = 2.08, SD = 0.96, *p* = 0.008), and was sustained until the end of the experiment (M = 2.09, SD = 0.98, *p* = 0.004). There were no other main or interaction effects (*p*s = 0.152–0.956). For Negative Affect, we found a significant main effect of Time [*F*_(1.92, 130.36)_ = 97.209, *p* < 0.001] and Suicide Risk [*F*_(3, 68)_ = 2.67, *p* = 0.05] but not Condition [*F*_(3, 68)_ = 2.404 *p* = 0.08). There was also a significant 2-way interaction between Time and Condition [*F*_(5.75, 130.36)_ = 3.94, *p* = 0.001). Specifically, compared to the Control condition (M = 1.39, SD = 0.56), the Combined condition had a significantly greater level of negative affect following the emotion induction task (M = 2.46, SD = 0.81, *p* = 0.034). There were no other interaction effects (*p*s = 0.40–0.77).

In both studies, the experimental manipulation procedures did not yield the intended effects. Specifically, the rumination inductions in both studies did not increase the intensity of the emotion generated by the emotion induction procedures. This limitation should be kept in consideration when interpreting the following results.

### Changes in Pain Responses^1^

#### Study 1

##### Pain Threshold

**Figure 1:**
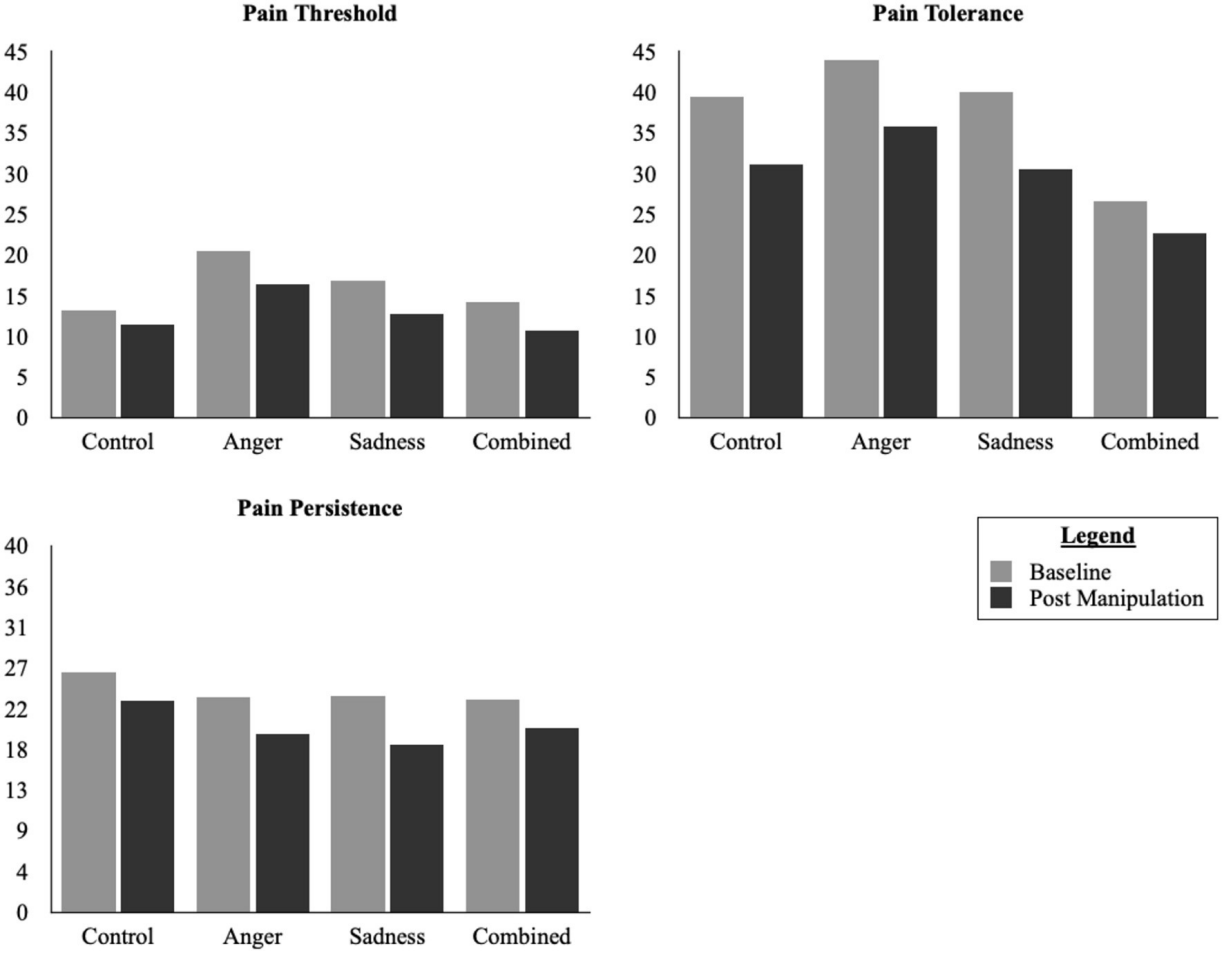
Changes in pain responses in Study 1.

Detailed information for the fixed and random effects found for pain responses in Study 1 are available in Table 4. Examining the model with both main and interaction effects, we found a significant main effect of Time [*F*_(1, 78.67)_ = 5.57, *p* = 0.02] but not Condition [*F*_(3, 80.57)_ = 0.32, *p* = 0.81], or Suicide Risk [*F*_(3, 80.58)_ = 0.87, *p* = 0.46]. There were no significant interaction effects (all *ps* > 0.43). When we removed the interaction terms, given that they did not improve the model, we similarly found a significant main effect of Time [*F*_(1, 92.90)_ = 18.45, *p* < 0.001) but not Condition [*F*_(3, 89.43)_ = 0.42, *p* = 0.94] or Suicide Risk [*F*_(3, 89.57)_ = 1.00, *p* = 0.40]. The results from a pairwise comparisons, using a Bonferroni correction to account for Family-Wise Error, indicated that there was a small but statistically significant decrease in Pain Threshold from Baseline (M = 17.41, SD = 17.47) and Post-Manipulation (M = 13.14, SD = 14.05, *d*_*av*_ = 0.27, *p* < 0.001, 95% CI = 5.16–14.05; See Figure 1).

**Table 4:** Study 1 fixed and random effects for pain responses.

	**Pain threshold**							
**Fixed effects**	**Parameter**	**Estimate**	**SE**	**df**	***t***	***p***	**95% confidence interval**	
							**Lower bound**	**Upper bound**
	Intercept	10.34	4.99	88.92	2.07	0.04	0.43	20.25
Condition	Control	−3.35	3.16	87.47	−1.06	0.29	−9.64	2.94
	Anger	−1.05	3.85	88.21	−0.27	0.79	−8.70	6.60
	Sadness	−1.06	3.12	87.15	−0.34	0.74	−7.26	5.14
	Combined	0*^b^*	–	–	–	–	–	–
Suicide risk	None	4.46	4.84	87.27	0.92	0.36	−5.16	14.08
	Ideators	−0.32	5.55	87.19	−0.06	0.95	−11.36	10.71
	Planners	−1.95	5.78	87.19	−0.34	0.74	−13.43	9.53
	Attemptors	0*^b^*	–	–	–	–	–	–
Time	Baseline	3.12	0.99	92.25	3.16	0.002	1.16	5.09
	Post–manipulation	0*^b^*	–	–	–	–	–	–
**Random effect**	**Parameter**	**Estimate**	**SE**		**Wald Z**	***p***	**95% confidence interval**	
							**Lower bound**	**Upper bound**
Repeated measures	Time	148.88	19.24		7.75	<0.001	115.58	191.79
**Pain tolerance**								
**Fixed effects**	**Parameter**	**Estimate**	**SE**	**df**	**t**	***p***	**95% confidence interval**	
							**Lower bound**	**Upper bound**
	Intercept	15.63	12.25	93.66	1.28	0.21	−8.70	39.96
Condition	Control	−1.25	7.72	89.86	−0.16	0.87	−16.58	14.07
	Anger	−5.46	9.41	91.28	−0.58	0.56	−24.14	13.22
	Sadness	−0.66	7.61	89.57	−0.09	0.93	−15.78	14.46
	Combined	0*^b^*	–	–	–	–	–	–
Suicide risk	None	17.92	11.91	92.59	1.50	0.14	−5.74	41.58
	Ideators	9.87	13.63	91.66	0.72	0.47	−17.20	36.94
	Planners	14.08	14.17	91.57	0.99	0.32	−14.06	42.23
	Attemptors	0*^b^*	–	–	–	–	–	–
Time	Baseline	9.51	2.21	93.91	4.29	<0.001	5.11	13.90
	Post-manipulation	0*^b^*	–	–	–	–	–	–
**Random effect**	**Parameter**	**Estimate**	**SE**		**Wald Z**	***p***	**95% confidence interval**	
							**Lower bound**	**Upper bound**
Repeated measures	Time	862.08	112.81		7.64	<0.001	667.05	1114.13
**Pain persistence**								
**Fixed effect**	**Parameter**	**Estimate**	**SE**	**df**	**t**	***p***	**95% confidence interval**	
							**Lower bound**	**Upper bound**
	Intercept	8.31	9.54	34.79	0.87	0.39	−11.06	27.68
Condition	Control	−0.23	5.51	32.37	−0.04	0.97	−11.45	10.99
	Anger	−4.84	6.80	32.86	−0.71	0.48	−18.69	9.00
	Sadness	0.03	5.47	36.11	0.01	1.00	−11.07	11.13
	Combined	0*^b^*	–	–	–	–	–	–
Suicide risk	None	9.14	9.05	34.24	1.01	0.32	−9.25	27.52
	Ideators	9.43	10.17	34.92	0.93	0.36	−11.23	30.09
	Planners	14.23	10.32	29.89	1.38	0.18	−6.86	35.32
	Attemptors	0*^b^*	–	–	–	–	–	–
Time	Baseline	4.87	1.90	77.10	2.57	0.01	1.09	8.65
	Post–manipulation	0*^b^*	–	–	–	–	–	–
**Random effect**	**Parameter**	**Estimate**	**SE**		**Wald Z**	***p***	**95% confidence interval**	
							**Lower bound**	**Upper bound**
Repeated measures	Time	346.99	121.27		2.86	0.004	174.91	688.36

b *Parameter was set to zero because it is redundant*.

##### Pain Tolerance

Examining the model with both main and interaction effects, we found a significant main effect of Time [*F*_(1, 79.68)_ = 5.62, *p* = 0.020] but not Condition [*F*_(3, 81.59)_ = 0.32, *p* = 0.81], or Suicide Risk [*F*_(3, 81.60)_ = 0.84, *p* = 0.47]. There were no significant interaction effects (all *ps* > 0.93). When we removed the interaction terms, given that they did not improve the model, we similarly found a significant main effect of Time [*F*_(1, 3.91)_ = 18.44, *p* < *0.0*01] but not Condition [*F*_(3, 90.44)_ = 0.13, *p* = 0.95] or Suicide Risk [*F*_(3, 90.58)_ = 0.98, *p* = 0.41). The results from a pairwise comparisons, using a Bonferroni correction to account for Family-Wise Error, indicated that there was a small but statistically significant in Pain Tolerance from Baseline (M = 41.11, SD = 30.82) and Post-Manipulation (M = 32.43, SD = 28.44, *d*_*av*_ = 0.29, *p* < 0.001, 95% CI = 5.11–13.91; See Figure 1).

##### Pain Persistence

Examining the model with both main and interaction effects while controlling for Pain Threshold, we found no significant main effect of Time [*F*_(1, 55.21)_ = 1.41, *p* = 0.24), Condition [*F*_(3, 56.66)_ = 0.38, *p* = 0.77], or Suicide Risk [*F*_(3, 41.38)_ = 0.55, *p* = 0.65]. There were also no significant interaction effects (all *ps* > 0.25). When we removed the interaction terms for the model, however, we found a significant main effect of Time [*F*_(1, 77.10)_ = 6.60, *p* = 0.01] but not Condition [*F*_(3, 32.80)_ = 0.23, *p* = 0.88], or Suicide Risk [*F*_(3, 30.34)_ = 0.65, *p* = 0.59]. The results from a pairwise comparisons, using a Bonferroni correction to account for Family-Wise Error, indicated that there was a small but statistically significant decrease in Pain Persistence from Baseline (M = 24.14, SD = 25.01) and Post-Manipulation (M = 20.76, SD = 22.65, *d*_*av*_ = 0.14, *p* = 0.01, 95% CI = 1.09–8.65; See Figure 1).

#### Study 2

##### Pain Threshold

**Figure 2:**
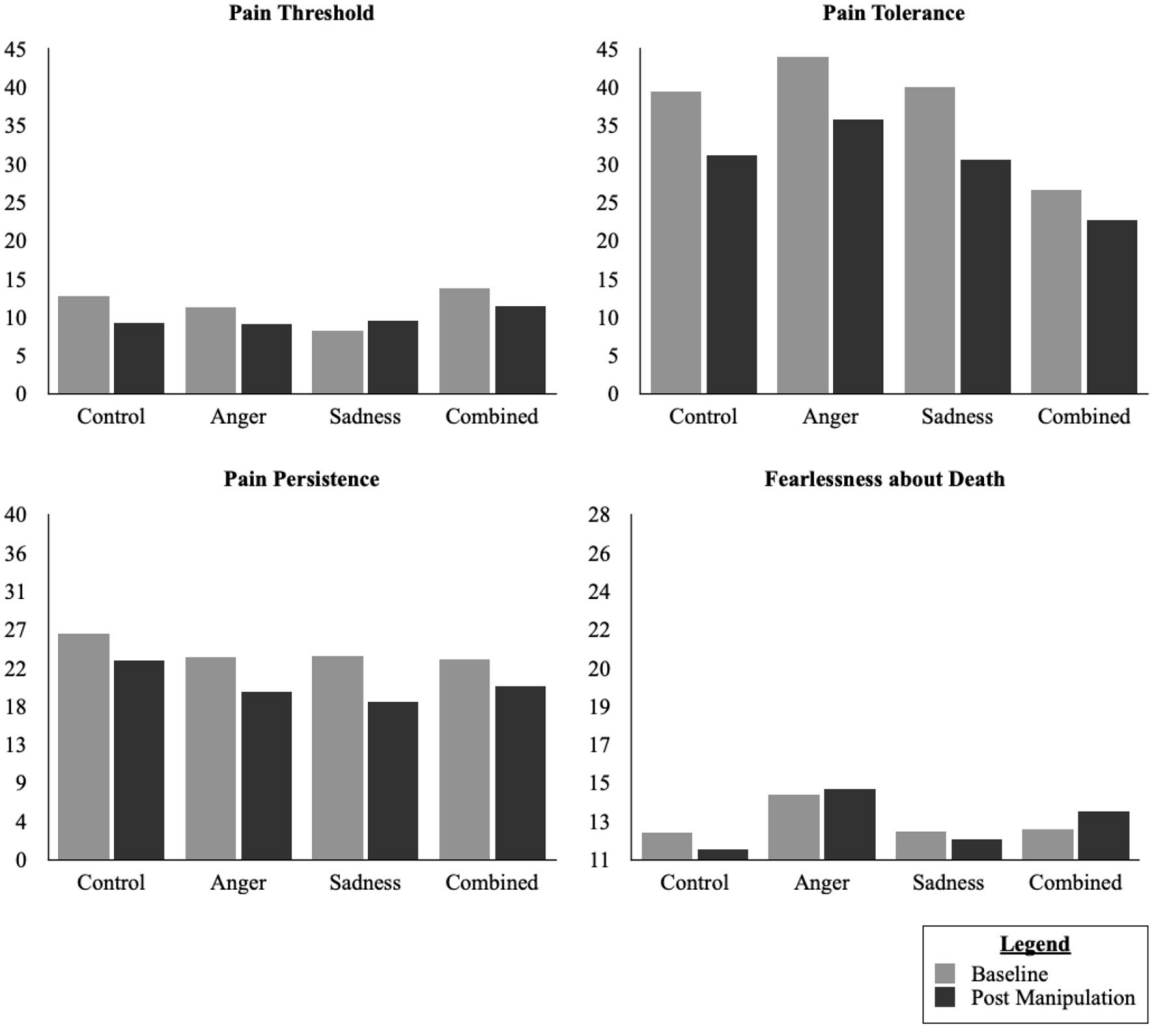
Changes in pain responses and fearlessness of death in Study 2.

Detailed information for the fixed and random effects found for pain responses in Study 1 are available in Table 5. Examining the model with both main and interaction effects, we found no significant main effect of Time [*F*_(1, 49.87)_ = 1.95, *p* = 0.17], Condition [*F*_(3, 51.86)_ = 0.66, *p* = 0.58], or Suicide Risk [*F*_(3, 51.91)_ = 0.17, *p* = 0.92). There were no significant interaction effects (all *ps* > 0.59). When we removed the interaction terms for the model, however, we found a significant main effect of Time [*F*_(1, 63.41)_ = 4.02, *p* = 0.05] but not Condition [*F*_(3, 59.42)_ = 0.52, *p* = 0.67] or Suicide Risk [*F*_(3, 59.75)_ = 0.29, *p* = 0.83]. The results from a pairwise comparisons, using a Bonferroni correction to account for Family-Wise Error, indicated that there was a small but statistically significant decrease in Pain Threshold from Baseline (M = 11.45, SD = 7.94) and Post-Manipulation (M = 9.74, SD = 7.47, *d*_*av*_ = 0.22, *p* = 0.05, 95% CI = 0.01–3.25; See Figure 2).

**Table 5:** Study 2 fixed and random effects for pain responses.

**Pain threshold**								
**Fixed effects**	**Parameter**	**Estimate**	**SE**	**df**	**t**	***p***	**95% confidence interval**	
							**Lower bound**	**Upper bound**
	Intercept	8.85	3.87	61.69	2.29	0.03	1.12	16.59
Condition	Control	−1.28	2.60	60.02	−0.49	0.62	−6.48	3.92
	Anger	−1.51	2.92	59.82	−0.52	0.61	−7.36	4.34
	Sadness	−3.27	2.72	59.69	−1.20	0.24	−8.71	2.18
	Combined	0*^b^*	–	–	–	–	–	–
Suicide risk	None	2.37	3.56	61.37	0.67	0.51	−4.74	9.49
	Ideators	3.58	3.93	60.68	0.91	0.37	−4.28	11.45
	Planners	2.92	4.95	60.12	0.59	0.56	−6.98	12.82
	Attemptors	0*^b^*		–	–	–	–	–
Time	Baseline	1.63	0.81	63.41	2.00	0.05	0.00	3.25
	Post-manipulation	0*^b^*	–	–	–	–	–	–
**Random effect**	**Parameter**	**Estimate**	**SE**		**Wald Z**	***p***	**95% confidence interval**	
							**Lower bound**	**Upper bound**
Repeated measures	Time	148.88	19.24		6.40	<0.001	45.13	83.24
**Pain tolerance**								
**Fixed effects**	**Parameter**	**Estimate**	**SE**	**df**	**t**	***p***	**95% confidence interval**	
							**Lower Bound**	**Upper Bound**
	Intercept	36.66	10.77	60.75	3.40	<0.001	15.12	58.21
Condition	Control	−3.92	7.26	59.75	−0.54	0.59	−18.43	10.60
	Anger	−4.63	8.17	59.61	−0.57	0.57	−20.98	11.71
	Sadness	−7.23	7.60	59.53	−0.95	0.35	−22.44	7.99
	Combined	0*^b^*	–	–	–	–	–	–
Suicide risk	None	−9.15	9.91	60.63	−0.92	0.36	−28.97	10.67
	Ideators	−4.31	10.97	60.18	−0.39	0.70	−26.26	17.64
	Planners	5.66	13.82	59.80	0.41	0.68	−21.98	33.31
	Attemptors	0*^b^*	–	–	–	–	–	–
Time	Baseline	6.95	1.74	62.96	4.00	<0.001	<0.001	10.42
	Post-manipulation	0*^b^*	–	–	–	–	–	–
**Random effect**	**Parameter**	**Estimate**	**SE**		**Wald Z**	***p***	**95% confidence interval**	
							**Lower bound**	**Upper bound**
Repeated measures	Time	446.08	73.94		6.03	<0.001	322.35	617.31
**Pain persistence**								
**Fixed effects**	**Parameter**	**Estimate**	**SE**	**df**	**t**	***p***	**95% confidence interval**	
							**Lower bound**	**Upper bound**
	Intercept	29.27	7.72	42.51	3.79	<0.001	13.69	44.85
Condition	Control	−1.95	5.35	53.01	−0.36	0.72	−12.68	8.78
	Anger	−1.80	5.96	49.42	−0.30	0.76	−13.78	10.17
	Sadness	−3.69	5.54	48.89	−0.67	0.51	−14.82	7.45
	Combined	0*b*	0*^b^*	–	–	–	–	–
Suicide risk	None	−14.40	7.08	41.81	−2.03	0.05	−28.70	−0.11
	Ideators	−9.54	7.84	41.40	−1.22	0.23	25.37	6.30
	Planners	0.35	9.92	44.74	0.04	0.97	−19.64	20.34
	Attemptors	0*^b^*	0*^b^*	–	–	–	–	–
Time	Baseline	5.47	1.63	55.93	3.35	=0.001	2.20	8.75
	Post-manipulation	0*^b^*	0*^b^*	–	–	–	–	–
**Random effect**	**Parameter**	**Estimate**	**SE**		**Wald Z**	***p***	**95% confidence interval**	
							**Lower Bound**	**Upper Bound**
Repeated measures	Time	231.14	50.72		4.56	<0.001	150.35	355.33

b *Parameter was set to zero because it is redundant*.

##### Pain Tolerance

Examining the model with both main and interaction effects, we found a significant main effect of Time [*F*_(1, 49.56)_ = 9.39, *p* = 0.004] but not Condition [*F*_(3, 52)_ =0.91, *p* = 0.44], or Suicide Risk [*F*_(3, 52.03)_ = 0.34, *p* = 0.80). There were no significant interaction effects (all *ps* > 0.10). When we removed the interaction terms we similarly found a significant main effect of Time [*F*_(1, 62.96)_ = 15.99, *p* < 0.001] but not Condition [*F*_(3, 59.35)_ = 0.30, *p* = 0.82] or Suicide Risk [*F*_(3, 59.57)_ = 0.96, *p* = 0.42]. The results from a pairwise comparisons, using a Bonferroni correction to account for Family-Wise Error, indicated that there was a small but statistically significant decrease in Pain Tolerance from Baseline (M = 33.02, SD = 22.93) and Post-Manipulation (M = 26.14, SD = 18.80, *d*_*av*_ = 0.33, *p* < 0.001, 95% CI = 3.48–10.42; See Figure 2).

##### Pain Persistence

Examining the model with both main and interaction effects while controlling for Pain Threshold, we found a significant main effect of Time [*F*_(1, 43.42)_ = 7.62, *p* = 0.008] but not Condition [*F*_(3, 34.61)_ = 0.89, *p* = 0.46] or Suicide Risk [*F*_(3, 33.60)_ = 1.28, *p* = 0.30]. There were also no significant interaction effects (all *ps* > 0.10). When we removed the interaction terms for the model we similarly found a significant main effect of Time [*F*_(1, 55.93)_ = 11.24, *p* = 0.001] but not Condition [*F*_(3, 46.87)_ = 0.15, *p* = 0.93], or Suicide Risk [*F*_(3, 45.61)_ = 2.54, *p* = 0.07]. The results from a pairwise comparisons, using a Bonferroni correction to account for Family-Wise Error, indicated that there was a small but statistically significant decrease in Pain Persistence from Baseline (M = 21.83, SD = 18.64) and Post-Manipulation (M = 16.77, SD = 14.29, *d*_*av*_ = 0.31, *p* = 0.001, 95% CI = 2.20–8.75; See Figure 2).

#### Indirect Effects Analysis

In Study 2, we then examined the role of arousal, measured by average Heart Rate (HR) on the relationship between Condition and changes in Suicide Capability. Using Model 4 in PROCESS (Hayes, 2013) with 10,000 bootstrapped samples, we found a significant direct effect of condition [*F*_(3, 52)_ = 3.09, *R*^2^ = 0.15, *p* = 0.04] on change in HR from the first cold pressor task to the second cold pressor task. Specifically, the Combined condition (B = 5.27, SE = 2.59, *p* = 0.05) but not the Anger Only or Sadness Only conditions (all *p*s > 0.19) demonstrated a significantly greater change in heart rate between the first and second cold pressor task compared to the Control condition. There were, however no significant direct effects of Condition or Heart Rate on changes in pain threshold [*F*_(4, 51)_ = 2.32, *p* = 0.07], pain tolerance [*F*_(4, 51)_ = 1.62, *p* = 0.18], and pain persistence [*F*_(4, 51)_ = 0.72, *p* = 0.58]. Although these models were not significant, we did notice a significant relative direct effect of Condition on pain threshold in the Sadness Only condition (B-6.35, SE = 2.22, *p* = 0.006). Unsurprisingly, 95% Confidence Intervals did not indicate the presence of significant indirect effects of Condition on changes in pain threshold, pain tolerance, and pain persistence through changes in heart rate. In regards to fearlessness of death, we found no significant direct effect between Condition and Changes in Heart Rate [*F*_(3, 68)_ = 2.24, *R*^2^ = 0.09, *p* = 0.09]. We also found no significant direct effect between Condition or Heart Rate on changes in fearlessness of death [*F*_(4, 67)_ = 1.70, *R*^2^ = 0.09, *p* = 0.16] but we did notice a significant relative direct effect of Condition on changes in fearlessness of death in the Combined condition only (B = 2.34, SE = 0.94, *p* = 0.02). There was no significant indirect effects of Condition on changes fearlessness of death through changes in heart rate.

When we compared the difference of change in HR from baseline to the first cold pressor task and baseline to the second cold pressor task, we found a significant direct effect of Condition [*F*_(3, 52)_ = 3.09, *R*^2^ = 0.15, *p* = 0.04]. Specifically, compared to the Control condition, the Combined condition (B = 5.27, SE = 2.59, *p* = 0.05) but not the Anger Only or Sadness Only conditions (all *p*s > 0.19) demonstrated a significant difference between change in HR from baseline to the first cold pressor task and baseline to the second cold pressor task. This difference in HR did not have any direct effects on pain threshold [*F*_(4, 51)_ = 2.32, *R*^2^ = 0.15 *p* = 0.07], pain tolerance [*F*_(4, 51)_ = 1.62, *R*^2^ = 0.11 *p* = 0.18], or pain persistence [*F*_(4, 51)_ = 0.72, *R*^2^ = 0.05, *p* = 0.58]. We did, however, observe a significant relative direct effect of Condition on changes in pain threshold in the Sadness Only Condition (B = 6.35, SE = 2.22, *p* = 0.006). There were no significant indirect effects of Condition on changes fearlessness of death through changes in heart rate (all *p*s > 0.09).

## Discussion

This study sought to experimentally test the differential effects of laboratory-induced rumination in the context of anger vs. sadness on changes in suicide capability. Furthermore, suicide risk was examined as a potential factor moderating the aforementioned relationships. Contrary to our hypotheses, we observed small decreases in all three pain variables following the experimental manipulations. These changes, however, did not appear to be related to rumination type or suicide risk. There are several possible explanations for these findings. First, past studies have suggested that individuals may be willing to persist through pain in service of achieving a desired goal (e.g., emotional relief or suicide; Anestis et al., 2012). In this study, however, participants received no incentive for persisting through both cold pressor tasks and thus participants may have been inadvertently motivated to end the cold pressor task early such that they can flee the negative affect generated from the emotion and rumination inductions. That being said, providing an incentive for participants to persist through a cold pressor or other pain tolerance task may enhance its accuracy as a proxy for measuring pain persistence and tolerance in the context of self-injurious and suicidal behavior.

Second, these findings appear to be consistent with findings from past studies that suggest individuals with a low baseline capability may react differently to emotional experiences compared to those with a high baseline capability (Ribeiro et al., 2014a; Law et al., 2015). Specifically, individuals who have a high baseline capability or regularly engages in non-suicidal self-injury may be more willing to tolerate and persist through pain and distress while their counterparts with low baseline capability may be more inclined to engage in behaviors that allows them to escape from pain and distress. We also found no significant differences in how suicide capability changed when comparing individuals who were asked to ruminate on high arousal emotions to those who were asked to ruminate on low arousal emotions. Although this may be attributable rumination induction's failure to amplify the emotions generated in the emotion induction. As previously mentioned, the experimental manipulation procedures did not yield the intended effects. Specifically, based on current theories and past studies, it was expected that rumination would increase the intensity of the emotion generated by the emotion induction procedures. In this study, however, the rumination induction failed to amplify the emotions and instead decreased negative affect and instead the greatest level of negative affect was found after the emotion induction and its intensity decreased following the rumination induction procedure. Both the emotion induction and rumination induction protocols, however, were selected due to their ability in past studies to elicit the expected emotional effects when compared to control and alternative conditions (Pitman et al., 1987; Rusting and Nolen-Hoeksema, 1998). Unfortunately, past studies using this combination of emotion and rumination induction procedures did not assess for changes in emotion between the two induction procedures (Law and Chapman, 2015). In this study, the addition of a measure of subjective emotional state between the two tasks may have decreased the effect of the combined emotion and rumination inductions. As such the anger and sadness rumination induced in our laboratory did not mimic past studies that have demonstrated success in using the combination of the emotion and rumination induction protocols and may not be the same as anger and sadness rumination as it occurs in a natural setting. Moreover, the experience of negative emotions may be characterized by mixed emotions. Asking participants to ruminate upon anger without sadness and sadness without anger may have resulted in a less ecologically valid representation of rumination in negative emotional experiences. Another potential factor that may have contributed to this decrease in negative emotions in between the two experimental manipulations may be the presentation of the emotion and rumination induction procedures. The emotion induction was personalized and presented with audio instructions while the rumination induction was generic and only presented as a series of slides that participants were asked to read. This difference may have impacted the participants' level of immersion in the task. It may be beneficial for future studies to consider presenting both emotion and rumination inductions using an audio format or combining the emotion and rumination induction tasks by injecting prompts for ruminative thinking into the participant's personalized scripts.

We also did not find a significant effect of suicide risk on changes in suicide capability following the experimental manipulations. When we examined the role of arousal, measured by average HR on changes in suicide capability, we found that rumination in the context of anger and sadness combined led to greater changes in HR between the first and second cold pressor task. These changes, however, did not translate into changes in suicide capability as measured by pain threshold, pain tolerance, pain persistence, and fearlessness about death. Given that a small proportion of participants in both studies reported a history of suicide attempts and/or ideation, the ability to detect the potential moderating role of suicide risk on rumination and pain experiences may have been obstructed. Accordingly, it would be important to replicate this study in a clinically relevant sample to better understand the how rumination may impact state changes in pain experiences in individuals high at risk for suicide. Suicide risk in this study was also determined solely on the presence of suicidal ideation and did not take into account other known indicators of elevated suicide risk such as tendency to cope using painful and provocative behaviors such as NSSI, the quality of an individual's suicidal ideation, the availability of a plan and means for suicide, and past history of suicide attempts (Chu et al., 2015). As such, future studies would benefit from using a more systematic assessment of suicide risk that takes into account other empirically determined factors contributing to an elevated risk for suicide.

There are several other limitations that warrant caution in the interpretation and generalization of these findings. Given that our results did not support our hypotheses, the models that were specified may not have been correct. The specific act of ruminating on an emotion may not be a factor that meaningfully contributes to changes in pain experiences. Rather, it may be the emotional experience, and its intensity, that drives the mechanisms leading to changes in the ability to tolerate pain (Carter et al., 2002). Rumination is also a coping method often used as a means to avoid the direct experience of emotions (Nolen-Hoeksema et al., 2008). Thus, the rumination induction may have provided participants with the opportunity to avoid experiencing the emotion generated in the emotion induction. Alternatively, perhaps the secondary emotions and behaviors born out of rumination such as self-blame or shame (Law and Chapman, 2015) are more salient than rumination at influencing changes in pain threshold, tolerance, and persistence. Additionally, we were also unable to measure changes in fearlessness of death given that the ACSS-FAD demonstrated poor internal consistency across all time points. It is possible that physical aspects of suicide capability (e.g., pain responses) are more stable while affective aspects of suicide capability (e.g., fearlessness) are more dynamic. Unfortunately, we were unable to determine if this is indeed the case. As such, future studies that wish to test for changes in fearlessness about death may want to consider using self-report or behavioral measures other than the ACSS-FAD. Finally, the study was lacking in diversity given that participants for both studies were largely white, heterosexual, cisgender females. Therefore, replication of this study in diverse samples will be needed to determine if our findings are generalizable.

Overall, this study represents a novel contribution to existing research on rumination and suicide risk by examining potential mechanisms by which rumination can facilitate the transition of suicidal ideation to the act of making a suicide attempt. Although the hypotheses of this study were largely unsupported, these findings offer an alternate way of conceptualizing pain experiences as being malleable and not simply stable traits. Ultimately, these findings serve as a conceptual and methodological springboard for additional research to examine possible factors that may contribute to acute changes in suicide capability that may enable an individual to engage in suicidal behavior. By understanding how cognitive and emotional factors interface with the capability for suicide, we may be able to generate the information and knowledge required to develop or refine existing interventions that can effectively reduce suicide risk by decreasing an individual's ability to make a lethal suicide attempt.

## Data Availability Statement

The datasets presented in this study can be found in online repositories. The names of the repository/repositories and accession number(s) can be found at: https://osf.io/8fyaj/?view_only=958c9630240f4d1588f551cd7875e41d.

## Ethics Statement

The studies involving human participants were reviewed and approved by the Institutional Review Board at The University of Southern Mississippi where the project was conducted as part of the first author's master's thesis and doctoral dissertation. The patients/participants provided their written informed consent to participate in this study.

## Author Contributions

KL designed, coordinated, conducted both studies under the supervision, cleaned and analyzed the data, wrote the manuscript, and prepared the tables and figures. MA provided supervision on consultation on the project which was conducted as part of the KL's master's thesis and doctoral dissertation and provided critical review of the manuscript. All authors contributed to the article and approved the submitted version.

## Conflict of Interest

The authors declare that the research was conducted in the absence of any commercial or financial relationships that could be construed as a potential conflict of interest.

## Publisher's Note

All claims expressed in this article are solely those of the authors and do not necessarily represent those of their affiliated organizations, or those of the publisher, the editors and the reviewers. Any product that may be evaluated in this article, or claim that may be made by its manufacturer, is not guaranteed or endorsed by the publisher.

## Footnotes

^1^Including trait-like levels of rumination into the model as a covariate did not change the results.
